# Adrenal adrenoceptors in heart failure

**DOI:** 10.3389/fphys.2014.00246

**Published:** 2014-07-07

**Authors:** Claudio de Lucia, Grazia D. Femminella, Giuseppina Gambino, Gennaro Pagano, Elena Allocca, Carlo Rengo, Candida Silvestri, Dario Leosco, Nicola Ferrara, Giuseppe Rengo

**Affiliations:** ^1^Department of Medical Translational Sciences, University of Naples Federico IINaples, Italy; ^2^Salvatore Maugeri Foundation, IRCCS, Scientific Institute of Telese TermeTelese Terme, Italy

**Keywords:** heart failure, adrenergic system, GRK2, adrenal gland, catecholamine, β-adrenergic receptor, functional recovery

## Abstract

Heart failure (HF) is a chronic clinical syndrome characterized by the reduction in left ventricular (LV) function and it represents one of the most important causes of morbidity and mortality worldwide. Despite considerable advances in pharmacological treatment, HF represents a severe clinical and social burden. Sympathetic outflow, characterized by increased circulating catecholamines (CA) biosynthesis and secretion, is peculiar in HF and sympatholytic treatments (as β-blockers) are presently being used for the treatment of this disease. Adrenal gland secretes Epinephrine (80%) and Norepinephrine (20%) in response to acetylcholine stimulation of nicotinic cholinergic receptors on the chromaffin cell membranes. This process is regulated by adrenergic receptors (ARs): α2ARs inhibit CA release through coupling to inhibitory Gi-proteins, and β ARs (mainly β2ARs) stimulate CA release through coupling to stimulatory Gs-proteins. All ARs are G-protein-coupled receptors (GPCRs) and GPCR kinases (GRKs) regulate their signaling and function. Adrenal GRK2-mediated α2AR desensitization and downregulation are increased in HF and seem to be a fundamental regulator of CA secretion from the adrenal gland. Consequently, restoration of adrenal α2AR signaling through the inhibition of GRK2 is a fascinating sympatholytic therapeutic strategy for chronic HF. This strategy could have several significant advantages over existing HF pharmacotherapies minimizing side-effects on extra-cardiac tissues and reducing the chronic activation of the renin–angiotensin–aldosterone and endothelin systems. The role of adrenal ARs in regulation of sympathetic hyperactivity opens interesting perspectives in understanding HF pathophysiology and in the identification of new therapeutic targets.

## Background

The sympathetic nervous system (SNS) is part of the autonomic nervous system and its activation was described by Cannon as “the fight or flight response” (Cannon, 1963). In the clinical setting, sympathetic nervous activity (SNA) can be evaluated by the analysis of plasmatic or urinary catecholamine concentrations but this estimation is variable depending on hormonal and other intra-individual adjustments, as well as on several factors such as glycemia, physical or psychological stress, and drugs. In recent years, many methods have been proposed for the assessment of SNA and, among these, microneurography and heart rate variability (HRV) are the most commonly used. Microneurography allows for a direct evaluation on electrical transmission in subcutaneous sympathetic nerves but it is not applicable for studies of large number of patients.

HRV analysis is spreading as a non-invasive technique for the evaluation of the autonomic nervous system influence on heart rate in various diseases and alterations in HRV have been shown to represent an independent predictor of mortality after myocardial infarction (Amadi et al., 1995). HRV gives an estimation on how the cardiac equilibrium between parasympathetic and sympathetic systems influences heart rate studying variations in cardiac frequencies.

Sympathetic hyperactivity has been recognized as a peculiar feature of several cardiovascular diseases as atherosclerosis, heart failure (Leimbach et al., 1986; Lymperopoulos et al., 2013), hypertension (Grassi, 1998), and syncope (Zysko et al., 2007).

Furthermore, sympathetic overdrive is associated also with non-cardiovascular pathologies: hyperglycemia and diabetes mellitus (Huggett et al., 2003), obesity and metabolic syndrome (MS) (Grassi et al., 2007), obstructive sleep apnea (Narkiewicz and Somers, 1997), and renal disease (Masuo et al., 2010).

Therefore some authors hypothesized a close connection between the components of the metabolic syndrome and augmented sympathetic activity suggesting a role of this latter in syndrome's establishment or progression (Esler et al., 2006). It is important to emphasize that most of SNA-related diseases, including HF, MS, and hypertension are major causes of morbidity and mortality worldwide. Sympathetic hyperactivity leads to an increase in arterial blood pressure and it is known to cooperate to the establishment and development of essential hypertension (Smith et al., 2004) through alterations in structural components of vessels and cardiac tissue with dysfunctional consequences.

HF is a chronic clinical syndrome characterized by the reduction in left ventricular (LV) function with the inability to adequately pump blood, maintain tissue perfusion, and support physiological functions. This leading disease represents one of the most important causes of morbidity and mortality worldwide (Go et al., 2014). Despite considerable advances in pharmacological treatment, HF represents a severe clinical and social burden.

During HF, several neurohormonal mechanisms get triggered in order to maintain cardiac output. The most important among these neurohormonal mechanisms are the SNS overdrive characterized by elevated circulating Catecholamines (CAs) and the Renin-Angiotensin-Aldosterone System hyperactivity. Consequently, sympatholytic drugs such as beta-blockers, Angiotensin-converting enzyme inhibitors, Ang-II receptor blockers and mineralcorticoid receptor antagonists are a cornerstones for the treatment of HF disease by ameliorating cardiac function (CIBIS-II Investigators and Committees, 1999; Von Lueder and Krum, 2013). The increase in circulating levels of Epinephrine (Epi) and Norepinephrine (NEpi) is initially needed to compensate heart dysfunction, according to the fundamental Frank-Starling law of cardiac function. However, if the cardiac insult persists, this law can no longer work and the process progressively becomes maladaptive and conducts to decompensated phase of HF, adversely impacting the clinical outcomes (Cohn et al., 1984; Lymperopoulos, 2013).

Body's major source of CAs is the adrenal medulla, the central part of the adrenal gland, where the chromaffin cells secrete approximately 20% NEpi and 80% Epi (Lymperopoulos et al., 2007b). The adrenal gland obtains input from the SNS through pre-ganglionic fibers and can be compared to a specialized sympathetic ganglion but it has the peculiar characteristic to secrete neurohormones directly into the blood. Chromaffin cells are post-ganglionic sympathetic neurons that have lost part of their peculiar characteristics as axons and dendrites and secrete their hormones into the bloodstream by exocytosis (Haase et al., 2011). The existing link between SNA and heart pathophysiology is very inescapable and suggestive.

In particular, since 1984 it was clear that plasma concentration of NEpi was negatively associated with survival in heart failure patients and the augmented plasma concentrations led to higher mortality (Cohn et al., 1984).

Furthermore, sympathetic overdrive in HF determines higher risk of arrhythmias and left ventricular dysfunction contributing to worsen the prognosis of this disease (Kaye et al., 1995). In addition, this linkage is more evident when evaluating cardiac consequences in Pheochromocytoma (PCC). PCC is rare neuroendocrine tumor of the adrenal glands medulla arising from the chromaffin cells (in 20% from extra-adrenal abdominal paraganglion tissue) and secreting high levels of catecholamines. Pheochromocytoma is present in 0.1–1% of patients with hypertension (Anderson et al., 1994) and it is present in phosphorylates of these tumors are mainly due to augmented CAs, particularly NEpi: tachycardia and palpitations, hypertension, acute myocardial infarction, angina, arrhythmias, left ventricular dysfunction, heart failure, and pulmonary edema. However, some Epi- and Dopamine-secreting tumors can determinate hypotension or cardiogenic shock (Bergland, 1989). Uncommon cardiac manifestations are rhythm disturbances as ventricular tachycardia, ST-segment elevation, prolongation of the QT interval and T-wave modifications.

CAs bind to adrenergic receptors (ARs) that are the principal mediators of SNS effects. So far, nine mammalian AR subtypes are known: three α1-AR, three α2-AR, and three β-AR (Bylund et al., 1994). ARs are part of the G-protein-coupled receptors (GPCRs) superfamily, membrane receptors that activate heterotrimeric G-proteins after their ligand binding. G-proteins typically stimulate (Gs-proteins) or inhibit (Gi-proteins) the enzyme adenylyl cyclase (AC) or activate (Gq-proteins) phospholipase C (PLC) (Rengo et al., 2009a). These receptors are phosphorylated by the family of GPCR kinases (GRKs) that regulates their pathway and function (Davis and Johnson, 2011). Cardiac role of β ARs is the regulation of heart rate and contractility in response to CAs.

Stimulation of β 1ARs (the primary subtype present on cardiomyocytes) and partially of β 2ARs has inotropic, dromotropic, cronotropic, and lusitropic effects (Grossini et al., 2013).

β1ARs and β2ARs activates both Gs proteins (stimulatory G proteins); however, β2AR can switch its signaling from Gs to Gi proteins when is phosphorylated by PKA. In addition, β 1AR stimulation determinates cardiomyocyte apoptosis while β2AR has antiapoptotic cardiac effects in the heart (Rengo et al., 2012c; Lymperopoulos, 2013; Lymperopoulos et al., 2013; Salazar et al., 2013).

High CAs levels determinate structural alterations in the heart: focal myocardium necrosis and monocytic inflammation, increased collagen deposition and consequent interstitial fibrosis in the arterial wall and in the myocardium (Roghi et al., 2011). Norepinephrine can increase cardiac oxygen consumption and myocytes apoptosis with consequent left ventricular alteration and dilated cardiomyopathy (Prejbisz et al., 2011). CAs conduct to cardiomyopathy by GRK2-mediated downregulation of β-adrenergic receptors in the heart (β-AR) and augmented intracellular calcium concentrations resulting in decreased cardiac contraction (Kassim et al., 2008).

Accordingly, it was shown that cardiac GRK2 levels and activity were increased in end-stage human HF and heterozygous GRK2 knockout mice have augmented cardiac contractility and function (Iaccarino et al., 1998, 1999; Iaccarino and Koch, 1999; Rengo et al., 2012b). Furthermore, transgenic mice overexpressing cardiac GRK2, have decreased cardiac function due to an excessive βAR dysfunction and oppositely mice with cardioselective expression of βARKct showed improved cardiac contractility at baseline and isoprotenerol-induced (Koch et al., 1995). GRK2 enhancement determinates cell death in ischemic cardiomyocytes, and its inhibition by an inhibitory peptide (β ARKct) is cardioprotective. Recently, it has been demonstrated that GRK2 is able to localize in mitochondria but his role is controversial. Koch et al. recently showed that GRK2 has a cardiac pro-death function by mitochondrial localization in myocytes after ischemic stress while Fusco et al. demonstrated that mitochondrial GRK2 plays a protective role regulating ATP production (Fusco et al., 2012; Chen et al., 2013).

Elevated circulating CAs can determinate myocardial damage by enhancing the cardiac oxygen request and by increasing peroxidative and lipoperoxidative metabolism and consequent free radicals production (Radtke et al., 1975). Severe LV dysfunction occurs in few patients and it seems to be secondary to genetic polymorphisms of the β-adrenergic receptors that increase the propensity to develop cardiomyopathy (higher sensitivity to catecholamines) (Small et al., 2002).

## α_2_-adrenoceptors

The α2-ARs are inhibitory autoreceptors that inhibit further release of CAs in adrenergic nerves in the central and in the SNS, including the adrenal gland.

The predominant inhibitory role of α2ARs in the adrenal gland results clear when considering that PC12, a rat pheochromocytoma cell line typically used as neuronal cell model, does not express these receptors and secretes abnormal CAs quantity.

However, it has been discovered that different α-AR subtypes explicate their main action in diverse organs (Brede et al., 2003). Due to the absence of selective drugs for the three α2-ARs subtypes, gene deletion in animals or cells lacking α2-AR subtypes have been necessary to understand the real function of these different subtypes. α2A-AR and α2C-AR perform their role as autoreceptors on neurons of peripheral nerve terminals and in the heart, inhibiting NEpi release. In particular, α2A-AR inhibits hormones release at high stimulation frequencies whereas the α2C-subtype plays his role at lower levels of nerve activity. Anyway low- and high-frequency stimulations are both important for synapse regulation (Hein et al., 1999). α2C-ARs is also implicated in some brain functions as vigilance, attention, stress reaction, gait, and locomotion (Sallinen et al., 1999) and some renal functions as well as tonic renal vasoconstriction and inhibition of renin release (Michel and Rump, 1996). α2B-AR subtype is mainly expressed in the central SNS and in vascular smooth (role of vasoconstriction) cells (Link et al., 1996) and it is involved in embryonic growth probably because of his function in placental angiogenesis (Macdonald et al., 1997). Moreover, the discrepancy in secretion (noradrenergic and adrenergic) in different groups of chromaffin cells should be (Hein et al., 1999) connected to different α2-AR subtypes expression.

In addition, α2-AR subtypes seem to play a part in neuronal differentiation. For this purpose Taraviras et al. studied the cellular modifications after Epi stimulation in PC12 cells expressing only one of the different α2-AR subtypes. They found that Epi can induce a diverse neuronal differentiation in a subtype-dependent way. Particularly, PC12α2B- and PC12α2C-transfected cells presented evident Epi-induced differentiation showing neurofilaments typical of differentiated neurons while PC12α2A-transfected cells didn't need Epi for their differentiation. Furthermore, they have shown that mitogen-activated protein kinase (MAPK) and Akt activation are needed for α2-AR–dependent neuronal differentiation (Taraviras et al., 2002). All these findings suggest that α2-AR subtypes differential expression in neuronal or neuron-like cells can influence not only organ tissue-specificity but also embryonic evolution and cellular differentiation. Moreover, α2-ARs could exert their neurogenic effects via the NF-kB pathway. NF-kB phosphorylation and consequent degradation of IkBα is under β-arrestins (β-arrs) control opening new interesting scenarios (Luttrell and Lefkowitz, 2002; Bathgate-Siryk et al., 2014). The specific subtypes of α2-ARs prevailing in the adrenal glands are still unknown and it seems there could be a species-specificity. Particularly in mice's adrenal gland α2C-AR subtype is the most important, while α2A-AR seems to be the most represented in rats (Lymperopoulos et al., 2007a). Thus, different expression of α2-AR subtypes reflects diverse neurotransmitter secretion in peripheral nerves and adrenal gland.

It is known that the major source for plasma NEpi are peripheral sympathetic nerve terminals while for Epi is the adrenal gland. The role of α2-AR in this story was clear when Brede et al showed that mice lacking the α2C-AR have twice plasmatic Epi levels compared to wild-type, whereas mice lacking the α2A-AR subtype presented higher NEpi levels of NEpi than wild-type (Brede et al., 2003).

In human adrenal the situation is controversial: α2A-AR is the most expressed but some authors reported that α2C-AR is present, too (Berkowitz et al., 1994). It is important to emphasize that human α2-AR subtypes dysfunction/deletion can influence SNS activation and heart function.

Patients with heart failure carrying a variant of the α2C-adrenoceptor with less function (α2C-Del322–325) showed reduced cardiac function (measured by echocardiography and cardiac catheterization) than patients with intact α2-adrenoceptor (Brede et al., 2002). Moreover, α2C-Del322–325 polymorphism in healthy people led to increased SNA and circulating CAs levels during supine rest and an augmented pharmacologically-induced NEpi and Epi secretion. On the other hand, human α2B-Del301–303 (consisting in a deletion of three glutamic acids) led to impaired agonist-promoted receptor phosphorylation and desensitization. Nguyen et al, showed that in α2B-transfected PC12 cells, this deletion produces an increased inhibitory function against nicotine-induced CAs secretion suggesting that some polymorphisms can confer a favorable phenotype in increased SNA–associated diseases as HF and hypertension (Nguyen et al., 2011).

Hence, further studies on α2-AR subtypes should help researchers to better understand pathophysiology of major cardiovascular diseases and then personalize their therapy.

## Catecholamines secretion in adrenal gland

The adrenal medulla is mainly constituted of groups of adrenergic and noradrenergic chromaffin cells and in minor part of ganglionic neurons. CAs derive from the amino acid tyrosine and are the principal hormones underlying the fight-or-flight response. Catecholamines from chromaffin cells are secreted after acetylcholine stimuli (from sympathetic ganglia) and their exocytosis is regulated by numerous membrane receptors (Becherer et al., 2012). Most of these receptors are GPCRs (G-Protein coupled receptor) comprehending ARs that exert their function as autoreceptors. In particular, β ARs (primarily β 2 subtype) stimulate CAs secretion (facilitatory autoreceptors) while the α2ARs inhibit CA secretion (inhibitory autoreceptors) (Foucart et al., 1988). ARs signaling and function are regulated by the family of GPCR kinases (GRKs), whose role has been well studied in HF (Rengo et al., 2009b, 2012b, 2014; Lymperopoulos et al., 2012; Salazar et al., 2013).

Circulating CAs originate from two major sources in the body: the sympathetic nerve endings, which secrete NEpi, and the chromaffin cells of the adrenal medulla, that liberate Epi (principally) and NEpi after acetylcholine stimulation of the nicotinic cholinergic receptors (nAChRs). Chromaffin cells act as a post-ganglionic sympathetic neuron and secrete different quantity of CAs in basal or stress conditions. However, circulating CAs levels have significant intraindividual and interindividual variations particularly after stressors as surgery (Sager et al., 1988). Basal percentages of adrenal CAs production are: 80% Epi (adrenal gland medulla is the chief source of Epi) o and 20% NEpi (Lymperopoulos et al., 2007a). Thus, we can summarize that cardiac β-ARs were bound by either Epi—deriving from the adrenal gland—than NEpi—from local sympathetic nerve terminals and in minor part from adrenal medulla. Anyway, in adrenal medulla there are other receptors that promote CAs secretion: muscarinic cholinergic receptors (mAChRs) (Zaika et al., 2004), angiotensin II receptors (Armando et al., 2004), and histaminergic receptors (Wallace et al., 2002). Furthermore, it has been shown that adenosine receptors act as inhibitory autoreceptors, though their real role and expression are not completely clarified (Tseng et al., 2001). ARs, including α2-AR and β-AR, undergo agonist-dependent desensitization and downregulation. These processes imply reduced receptor response and increased internalization due to constant or repetitive agonist binding (Reiter and Lefkowitz, 2006).

In particular, after ligand stimulation, receptor is phosphorylated by GPCR kinases (GRKs), with the subsequent binding of β-arrs to the GRK-phosphorylated receptor. Consequently, β-arrs uncouple the receptor from its related G-proteins, preventing its further binding to G-proteins and leading to downregulation (Reiter and Lefkowitz, 2006; Lymperopoulos et al., 2009, 2011).

To date, GRK2, GRK3, and GRK5 are the most significant members among the GRKs because they are present ubiquitously in mammalian body (particularly in brain and cardiac tissue) and phosphorylate most of the GPCRs. Notably, GRK2 is upregulated in the heart and adrenal glands in HF and in vascular tissue during hypertension; strategies that inhibit or inactivate GRK2 in these diseases are very interesting for future human therapy (Gurevich et al., 2012).

It has been shown that human β 1- and β 2-ARs (*in vivo* and *in vitro*) and α2A- and α2B-ARs (*in vitro*) are phosphorylated by GRK2 but it isn't clear if α2C-AR is a GRK2 substrate, yet (Jewell-Motz and Liggett, 1996; Rengo et al., 2012c).

Besides, the role of GRK2 on α2C-ARs phosphorylation has been demonstrated in other species, prompting to similar hypothesis in humans (Lembo et al., 1999). Recently Cortez et al. showed that β 1-, β 2-, and β 3-ARs are expressed in cultured human adrenal chromaffin cells and in particular β 2- and β 3-ARs stimulation determinate CAs release and β 2- and β 3-antagonists counteract nicotine-induced CAs secretion (Cortez et al., 2012). CAs secretion by chromaffin cells is also strongly regulated by adrenal gland cortex. In the whole adrenal gland, the medulla and the cortex, though with a diverse embryological development, are strictly linked and crosstalk in anatomical and functional ways, influencing each other.

In particular, Glucocorticoids (GCCs), among the steroids secreted by the adrenal cortex, determinate a multitude of effects on medullary chromaffin cells. The steroids, binding their nuclear receptors, activate some transcriptional factors that increase CAs production and release, upregulate Tyrosine hydroxylase and activate an alternative splicing of phenylethanolamine N-methyltransferase, a key enzyme in the transformation of NEpi in Epi. Moreover, GCCs influence chromaffin cell differentiation and characterization, determining the acquirement of adrenergic phenotype, particularly for the cell groups adjacent to adrenal cortex (Hodel, 2001). In addition, recent studies on knockout mice (in particular for the 21-hydroxylase or for the Corticotropin releasing hormone receptor 1 genes) confirmed that GCCs stimuli is necessary for the acquisition of the adrenergic but not the noradrenergic phenotype. It is also striking that chromaffin cell products as NEpi, Epi, Dopamine, VIP and Serotonin can enhance steroidogenesis of cortical hormones (Aldosterone, Cortisol, Androstendione, Deoxycorticosterone) in a paracrine way (Haase et al., 2011).

Of note, Flugge et al. demonstrated that GCCs determinate diverse expression of α2A-and α2C-ARs in brain during chronic stress. This finding suggests that adrenal cortex hormones could influence not only the adrenergic/noradrenergic phenotype but also the adrenal αAR expression/function thus cooperating in sympathetic overdrive-related diseases (Flugge et al., 2003).

## Adrenal GRK2 and cardiovascular pathophysiology

**Figure 1:**
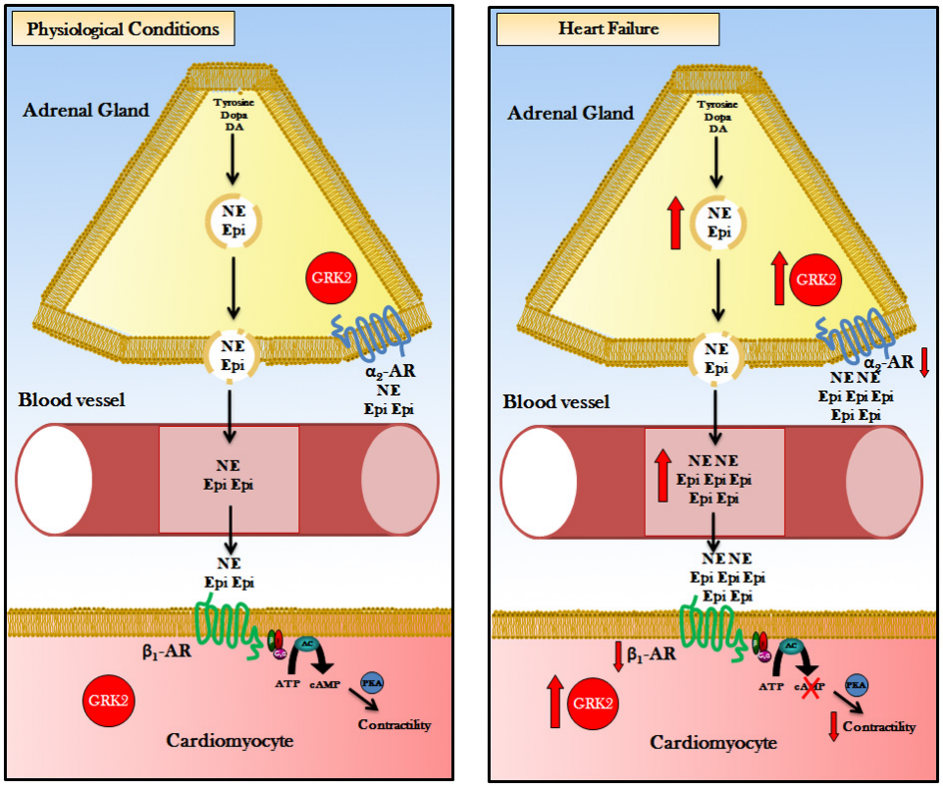
**Representation of the pathophysiologic role of GRK2 in adrenal CA-production/secretion: Body's major source of CAs is the adrenal medulla, the central part of the adrenal gland, where the chromaffin cells secrete approximately 20% NEpi and 80% Epi**. Physiological conditions: G-protein-coupled receptor kinase 2 (GRK2) regulates ARs: (1) in chromaffin cell of adrenal gland GRK2 phosphorylate α2ARs that exert a tonic sympathoinhibitory function. (2) in cardiomyocytes GRK2 phosphorylate β 1-AR regulate cardiac contractility by AC-PKA pathway activation. Heart Failure: G-protein-coupled receptor kinase 2 (GRK2) is upregulated in chromaffin cell and in cardiac myocyte. In the adrenal chromaffin cell, augmented GRK2 levels determinate an hyper-phosphorylation and desensitization of α2ARs, causing increased levels of Epi/NE production and secretion. Increasing in amounts of circulating CAs led to hyper-stimulation of β 1-AR and GRK2 overactivation. Cardiac GRK2 upregulation results in phosphorylation and desensitization/downregulation of β 1-ARs leading to reduction of contractility. Consequently, double inhibition of GRK2 (pharmacological or gene therapy) in the heart and in the adrenal gland could have impressive therapeutic effect in heart failure enhancing cardiac contractility and reducing plasmatic CAs levels. Acronyms: CAs, Catecholamines; DA, Dopamine; NE, Norepinephrine; Epi, Epinephrine; GRK2, G protein-coupled Receptor Kinase 2; ARs, Adrenergic Receptor; α2-AR, α2-Adrenergic Receptor; β 1-AR, β 1-Adrenergic Receptor; ATP, Adenosine Tri-Phosphate; AC, Adenylyl Cyclase; cAMP, cyclic Adenosine Mono-Phosphate; PKA, Protein Kinase A.

HF is characterized by elevated sympathetic tone with augmented levels of circulating and synaptic CAs. In the early phase of the disease increased SNA is an useful and compensatory mechanism to maintain cardiac output by increasing heart rate and cardiac contractility but, when β-ARs become disresponsive to CAs, this chronic stimulation determinates HF progression and its consequent detrimental systemic effects (Port and Bristow, 2001). Some studies in the last 10 years underline the critical inhibitory role of presynaptic α2-AR in peripheral nerve terminals and in adrenal medulla. This finding became clearer when mice with genetic deletions or knockout (KO) for α2-AR where studied. Particularly, α2A- or α2C-ARs KO mice that underwent HF after TAC-induced pressure overload presented an increase in circulating CAs with subsequently decreased cardiac parameters compared to control mice (Brede et al., 2002). In addition, double α2A/α2C-AR KO mice showed cardiomyopathy at 4 months of age, without surgery or other treatments (Brum et al., 2002). The crucial function of human α2-AR in HF development and progression was elucidated by studies on genetic polymorphism of this receptor. Small et al. demonstrated that α2CDel322–325 polymorphism is associated with high HF risk (Small et al., 2002; Davis and Johnson, 2011) probably because this variant was associated to increased α2-AR-related CAs secretion/outflow (as shown *in vitro*) (Small et al., 2000) and subsequent detrimental cardiotoxicity due to β-AR downregulation/desensitization. Our group demonstrated few years ago that adrenal hyperfunction is crucial for HF development and evolution (Lymperopoulos et al., 2007a). During HF there is an increase in CAs production, testified by enhanced tyrosine hydroxylase levels, and secretion (both NEpi and Epi) by hypertrophic adrenal glands (Figure 1). To better understand if this mechanism was peculiar of HF, we tested two different models of HF for etiology and pathology. Particularly, we evaluated rats that developed congestive HF 10 weeks after myocardial infarction induced by surgical ligation of left anterior descending coronary artery and transgenic mice with cardiac overexpression of sarcoplasmic reticulum calcium-binding protein calsequestrin that underwent progressive HF in a short time (3 months) and commonly died when they become 4 months old. In both our models of HF (independently from the reason that determinates this disease) we found adrenal GRK2-related α2-AR desensitization and downregulation that lead to enhanced circulating CAs levels. Furthermore, Schneider et al. demonstrated adrenal GRK2 upregulation in a model of cardiac hypertrophy due to pressure overload (obtained by TAC surgery), too. As expected, the degree of cardiac hypertrophy was significantly associated with adrenal weight and adrenal CAs production (Schneider et al., 2011). Of note, GRK2 increase results in α2-AR phosphorylation and subsequently in loss of inhibitory feedback (Figure 1). This ends up in the increase of Epi and NEpi release incisively contribute to SNS overdrive. The main function of adrenal GRK2 in sympathetic overactivity and consequent progression of HF became more evident when we tried to contrast GRK2 increase by direct adrenal injection of its inhibitor β ARKct in HF rats (this peptide is the C-terminal part of GRK2 that doesn't contain the phosphorylation portion but competes with GRK2 for G-proteins βγ subunits binding). In particular we used an Adenovirus codifying for β ARKct and 1 week after gene delivery we performed the *in vivo* and *in vitro* evaluations. β ARKct was able, by inhibiting GRK2, to restore α2AR membrane levels/function and subsequently have a sympatholytic effect lowering plasma CAs levels. This permits to counteract CA cardiotoxic effects by decreasing cardiac β-AR downregulation/desensitization and thus ameliorate heart dilatation and function as attested by echocardiography and *in vivo* cardiac hemodynamic.

Recently, we decided to investigate if GRK2 inhibition before HF onset can determinate any advantage in development and progression of this invaliding disease. For this purpose we used Cre/loxP technology to obtain tissue-specific GRK2 KO mice. In particular, GRK2 was deleted only in chromaffin cells of adrenal medulla by the use of mice expressing Cre recombinase under the control of the phenylethanolamine N-methyl transferase (PNMT) gene promoter (PNMT-driven GRK2 KO mice) (Lymperopoulos et al., 2010). PNMT is the enzyme that catalyses the trasformation of NEpi into Epi and this function is peculiar in chromaffin cells. According to our results, adrenal GRK2 pre-HF deletion allows for a significant attenuation of adrenal hypertrophy and reduction of *in vivo* plasmatic CAs in post-MI HF mice. Decreased systemic cathecolaminergic stimulation that is usually detrimental for HF establishment, determinates lower cardiac β-AR downregulation/desensitization (GRK2 decreasing-mediated), with a consequent better heart function and enhanced cardiac inotropic reserve. Significantly, the PNMT-driven GRK2 KO mice showed a characteristic basal phenotype: reduced CAs production (lower Thirosine Hydroxylase protein levels) and adrenal dimensions. All these findings suggest that GRK2 could be a significant adrenal trophic element in physiologic conditions and in HF in particular, being a crucial CAs production regulator (directly acting on biosynthetic enzymes or indirectly by β 2-AR mediated CAs secretion stimulation). In addition, our group has recently shown that adrenal GRK2 is also a physiological regulator of adrenal CAs production/secretion and thereby of SNA. In particular, in healthy rats, adrenal GRK2 adenovirus-mediated (Ad-GRK2) gene delivery led to increased plasmatic levels of Epi and NEpi whereas Ad-β ARKct adrenal gene transfer determined a significant decrease of the same levels. Of note, despite NEpi was only the 20% of the total CAs secreted by adrenal medulla, gene delivery influencing GRK2 activity is able to change its levels. These results were confirmed by *in vitro* chromaffin cells experiments that also showed, as expected, that physiological adrenal GRK2 action is α2-AR mediated (Lymperopoulos et al., 2008). Moreover, adrenal GRK2 has a significant role on beneficial sympatholytic effects of β-blockers and exercise training during HF (Rengo et al., 2010, 2012a; Femminella et al., 2013a). Importantly, training and beta-blocker therapy are know to have several biological effects and to improve survival in HF. Effectively, both these therapies led to a reduction of adrenal GRK2 levels/activity which conducts to decrease and normalization of CAs biosynthesis and production through restoration of α2-AR density/signaling. In our study on β-blockers effects on adrenal gland activity, we evaluated bisoprolol (a β 1-AR selective blocker) to exclude any involvement of facilitatory pre-synaptic β 2-ARs (see above). Of note, bisoprolol effects on left ventricular reverse remodeling preceded adrenal GRK2 downregulation and α2AR restoration. Consequently, these two treatments could exert a complementary neurohormonal action in contrasting the detrimental consequence of autonomic overdrive that affects HF patients. Furthermore, α2-AR dysfunction during HF may have important therapeutic implications because it could explain the failure of MOXSE and MOXCON trials (Swedberg et al., 2002). These trials were interrupted for the excessive mortality in the treated group and one of the possible explanations could be the dysfunction of α2-AR in adrenal medulla and peripheral nerve terminals that could not permit the drug to exert its beneficial consequences. Therefore, β-blockers and exercise training treatments taking advantage of their adrenal α2-ARs effects could potentially impact on moxonidine efficacy during HF.

However, the complete mechanism through which adrenal CAs overdrive occurs in HF is still unclear. In this regard some studies in dogs showed that bilateral adrenal denervation significantly reduced heart dysfunction after cardiac pressure overload (Womble et al., 1980).

Accordingly, it has recently been shown that unilateral denervation of the adrenal gland from the preganglionic cholinergic nerves, did not permit adrenal hypertrophy and rising of CAs production during cardiac pressure overload (Schneider et al., 2011). Hence, cholinergic innervation of the adrenal gland by nicotinic receptors and a Ca2++/calmodulin-dependent signaling is crucial to determinate adrenal hypertrophy, increase GRK2 levels and raise NEpi and Epi storage.

Of note, in isolated adrenal gland with undamaged splanchnic nerves, cholinergic stimulation caused release of cortisol and aldosterone (Ehrhart-Bornstein et al., 1995). These findings, together with the strict adrenocortical linkage (treated above), suggest that adrenal activation could be triggered by preganglionic cholinergic nerves stimulation through release of corticosteroids hormones.

## Conclusions

CAs levels are a powerful prognostic factor of morbidity and mortality in HF (Cohn et al., 1984). GRK2 has a multiorgan pivotal role: in adrenal medulla and in cardiac nerve terminals this kinase regulates NEpi/Epi production and secretion through α2-ARs, whereas in heart it mediates cardiac effects of CAs by β-ARs regulation. In particular, adrenal GRK2-dependent α2-AR dysregulation seems to be crucial in enhanced CAs secretion from the adrenal gland during HF, contributing to detrimental sympathetic cardiotoxic effects. Consequently, restoration of adrenal α2-AR signaling through the inhibition of GRK2 may be a novel sympatholytic therapeutic strategy for HF. Decreasing CAs levels would permit restoration of cardiac β-AR downregulation/desensitization via cardiac GRK2 downregulation and ameliorate some critical aspects of failing heart such as adverse remodeling, arrhythmias and cardiac arrest. Of note, several therapeutic strategies, as β-blockers and exercise training, can exert their beneficial effects on HF also by decreasing sympathetic overdrive through adrenal GRK2 inhibition (probably also in sympathetic nerve terminals).

Significantly, systemic GRK2 inhibition during HF might be impressive because of its well-known positive cardiac effects and its ability to thwart the chronic activation of the renin–angiotensin–aldosterone (GRK2 inhibition could counteract phosphorylation and desensitization of Angiotensin II receptor type 1) and endothelin (GRK2 inhibition could prevent endothelin-induced insulin resistance) systems (Rockman et al., 1996; Zolk et al., 1999; Anavekar and Solomon, 2005; Usui et al., 2005). Furthermore it is interesting that GRK2 inhibition could be obtained by both systemic administration of a pharmaceutical GRK2 inhibitor molecule (Piao et al., 2012) or by local (cardiac or eventually adrenal) and systemic gene therapy delivery (Zincarelli et al., 2008, 2010).

In addition, GRK2 inhibitors could be useful as adjunctive therapy in HF, thus reducing the dosage and consequently the adverse effects of β-blockers.

As discussed above, α2-AR agonists are able to increase α2-AR inhibitory activity and thus to determinate sympatholysis in HF due to peripheral and adrenal α2-AR downregulation/desensitization. Importantly, our group evidenced that the therapeutic effects of moxonidine on decreasing CAs *in vivo* in rats with HF were enhanced with GRK2 inhibition via adrenal gene therapy. Of note, this combined therapy led to lower Epi levels, a non-typical phenomenon for moxonidine alone (Lymperopoulos et al., 2007a).

Adrenal GRK2 inhibition could be also positive and valuable as a therapy for other diseases characterized by sympathetic hyperactivity as hypertension (Schlaich et al., 2004), hyperthyroidism (Foley et al., 2001), pheochromocytoma (Roghi et al., 2011) or some cognitive, and psychiatric disorders as depression (Hausberg et al., 2007; Femminella et al., 2013b).

To summarize, cardiac and adrenal GRK2 inhibition represents an important therapeutic target during HF. However, further studies would be necessary to better understand the underlying complete mechanism and to allow potential and innovative specific peptides or gene delivery techniques to become part of common HF therapy.

### Conflict of interest statement

The authors declare that the research was conducted in the absence of any commercial or financial relationships that could be construed as a potential conflict of interest.
